# Genetic structure and demographic history of the endangered tree species *Dysoxylum malabaricum* (Meliaceae) in Western Ghats, India: implications for conservation in a biodiversity hotspot

**DOI:** 10.1002/ece3.669

**Published:** 2013-08-06

**Authors:** Sofia Bodare, Yoshiaki Tsuda, Gudasalamani Ravikanth, Ramanan Uma Shaanker, Martin Lascoux

**Affiliations:** 1 Department of Ecology and Genetics, Evolutionary Biology Centre, Uppsala UniversityNorbyvägen 18D, 75236 Uppsala, Sweden; 2 Ashoka Trust for Research in Ecology and the EnvironmentRoyal Enclave, Srirampura, Jakkur Post, Bangalore, 560064, Karnataka, India; 3 School of Ecology and Conservation, University of Agricultural SciencesBangalore, 560065, Karnataka, India; 4 Department of Crop Physiology, University of Agricultural SciencesGKVK Bangalore, 560065, Karnataka, India

**Keywords:** Conservation genetics, *Dysoxylum malabaricum*, population demography, simple sequence repeats, Western Ghats

## Abstract

The impact of fragmentation by human activities on genetic diversity of forest trees is an important concern in forest conservation, especially in tropical forests. *Dysoxylum malabaricum* (white cedar) is an economically important tree species, endemic to the Western Ghats, India, one of the world's eight most important biodiversity hotspots. As *D. malabaricum* is under pressure of disturbance and fragmentation together with overharvesting, conservation efforts are required in this species. In this study, range-wide genetic structure of twelve *D. malabaricum* populations was evaluated to assess the impact of human activities on genetic diversity and infer the species’ evolutionary history, using both nuclear and chloroplast (cp) DNA simple sequence repeats (SSR). As genetic diversity and population structure did not differ among seedling, juvenile and adult age classes, reproductive success among the old-growth trees and long distance seed dispersal by hornbills were suggested to contribute to maintain genetic diversity. The fixation index (*F*_IS_) was significantly correlated with latitude, with a higher level of inbreeding in the northern populations, possibly reflecting a more severe ecosystem disturbance in those populations. Both nuclear and cpSSRs revealed northern and southern genetic groups with some discordance of their distributions; however, they did not correlate with any of the two geographic gaps known as genetic barriers to animals. Approximate Bayesian computation-based inference from nuclear SSRs suggested that population divergence occurred before the last glacial maximum. Finally we discussed the implications of these results, in particular the presence of a clear pattern of historical genetic subdivision, on conservation policies.

## Introduction

Forest fragmentation caused by changes in human land use is of primary concern for sustainability and conservation biology in terrestrial ecosystems across the Earth (Aguilar et al. 2006) and, especially, in many tropical countries that have been experiencing rapid population growth over the last decades (Abdullah and Nakagoshi 2007). Conservation of tropical trees is particularly important as they provide habitats and ecological niches for thousands of species (Hamilton 1999). As fragmentation restricts pollen and seed dispersal, it modifies gene flow and alters historical patterns of genetic subdivision (Murawski et al. 1994). Hence, anthropogenic landscape change and habitat fragmentation may threaten the genetic connectivity of many plant species and ultimately lead to their disappearance as isolated populations are at risk of losing genetic diversity that is critical to their long-term survival (Sork and Smouse 2006). Thus, the evaluation of the impact of fragmentation on genetic diversity of forest trees has been one of the main topics in forest conservation for many years (Murawski et al. 1994; Hamilton 1999; Bacles et al. 2006; Jump and Peñuelas 2006).

**Figure 1:**
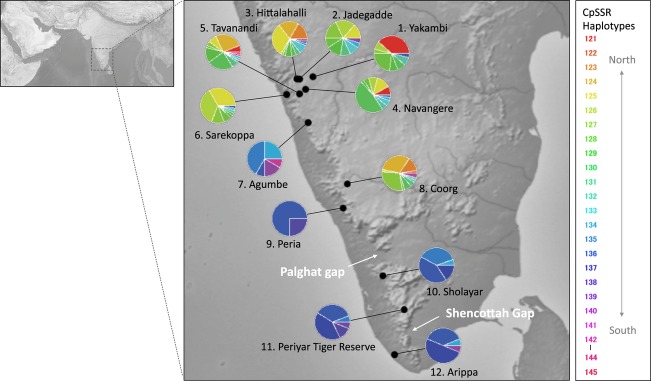
The location of the 12 *Dysoxylum malabaricum* populations and the distributions of chloroplast DNA haplotypes.

The Western Ghats region is a long mountainous massif (8–22°N, 73–77°E) that runs along the entire west coast of peninsular India (Fig. 1 Kodandapani et al. 2004). Together with Sri Lanka, the Western Ghats is one of the world's eight most important biodiversity hotspots based on exceptional endemism and conservation need (Myers et al. 2000). However, the Western Ghats faces severe threats from human disturbance due to deforestation, development activities, conversion of forests to plantations, and habitat fragmentation (Raman 2006), while natural reserves in the area are limited in size and fragmented by an intervening matrix of agricultural land and tree plantations (Kodandapani et al. 2004). Indeed, Menon and Bawa (1997) estimated that the natural vegetation of the Western Ghats has declined by 40% during the period 1920–1990, resulting in a fourfold increase in the number of fragments and an 83% reduction in size of surviving forest patches (Raman and Mudappa 2003). In the southern part of Western Ghats, Jha et al. (2000) detected a loss of 25.6% in forest cover over the period 1973–1995. One of the major causes of forest fragmentation in the Western Ghats is the spread of plantations, particularly, tea, coffee, and *Eucalyptus* (Raman 2006). Although India's net forest cover has actually increased since the 1990s due to agro-forestry plantations, social forestry, and mass afforestation (Ravikanth et al. 2000), the health of the native forests has actually decreased through reduction of canopy cover and forest density. Therefore, forest degradation and overexploitation of individual species is still an ongoing process in a large proportion of the existing forests (Rawat and Kishwan 2008), and it justifies conservation efforts for tree species of the Western Ghats. As genetic diversity of historical lineages cannot be recovered if it is lost (Moritz 2002), assessment of conservation genetics of species for which there is significant risk of diversity loss is essential to maintain their evolutionary integrity (e.g., Frankel 1974; Crandall et al. 2000; Moritz 2002). As the importance of biogeographical considerations in attempts to conserve populations of diverse organisms is also becoming increasingly clear (Whittaker et al. 2005), a wide-scale genetic survey of the target species complete range and the identification of the main historical lineages of the species area crucial first step.

Unfortunately only a handful of studies have examined genetic variation of plant species in the Western Ghats (Deshpande et al. 2001; Nageswara Rao et al. 2001, 2007; Padmini et al. 2001; Ravikanth et al. 2001, 2008; Bahulikar et al. 2004; Ramesha et al. 2007; Ismail et al. 2012), and none of them have examined wide-scale genetic structure and population demography of plant species. In particular, one could expect to find distinct population structure as the Western Ghats has two old (500 million years) geographical gaps in the southern part, the Palghat and Shencottah gaps (Robin et al. 2010). The former is a 30–40 km wide valley stretching from the west coast inwards at 11°N and is the largest disruption in this continuous mountain range while the latter is more narrow (7.5 km at 9°N). Although the age and origin of the Palghat gap is still controversial (D'Cruz et al. 2000), its impact on population genetic structure has been shown in three recent studies in elephants (Vidya et al. 2005), montane birds (Robin et al. 2010), and frogs (Nair et al. 2012). Other than that, very little is known on the impact of the topography of the Western Ghats on genetic structure, especially in plants, and it is not known whether these gaps could be considered as boundaries for conservation units of plant species.

White cedar (*Dysoxylum malabaricum* Bedd. [Meliaceae]) is a large canopy tree found in evergreen and semievergreen forests of 200–1200 m altitude in the Western Ghats. It grows to a height of 30–40 m or more, and 3–4 m girth (Shivanna et al. 2003). *D. malabaricum* is primarily an outbreeding species, but rare cases of selfing are also observed (Ismail et al. 2012). It is an economically important tree species endemic to the Western Ghats. Among many local tribes, *D. malabaricum* is a sacred tree used for medicinal, nutritional, commercial, and religious purposes (Kumar 2009). Its lustrous and sweet-scented wood is highly valued for various woodworks (Shivanna et al. 2003) and its fruits and wood are also harvested for use in traditional medicine. In fact, recent research has shown that compounds in *D. malabaricum* may be effective against malaria mosquitoes (Nathan et al. 2006). In the Western Ghats, forests are a patchwork of state, community, and privately owned land. *D. malabaricum* is protected by law in state-owned forests and may not be harvested there. In private forests it may be harvested by locals after permission from the Forest department, which also involves a fee, and they are harvested typically after they attain a girth of 180 cm (Menon and Balasubramanyan 2006). However, due to its high economic value, the natural range of *D. malabaricum* has been heavily fragmented (Shivanna et al. 2003). Although *D. malabaricum* has not yet been assessed by the IUCN Red List, it is has already been categorized as Endangered (EN) under the Indian National Threat Assessment using the same criteria as the IUCN (Ravikumar and Ved 2000). Regeneration is poor in this species, and a loss of juvenile individuals was prominent in a study conducted in Navangere, one of the northernmost populations (Shivanna et al. 2003; Y. Tsuda, pers. obs.). In line with this, another study in Coorg, in the central Western Ghats, detected almost no young adult individuals of *D. malabaricum* (S. A. Ismail, J. Ghazoul, G. Ravikanth, R. Uma Shaanker, C. G. Kushalappa, C. J. Kettle, submitted; Khan 2007). The absence of young individuals could be due to not only the overharvest of wood and fruit (Shivanna et al. 2003) but also to the combined impact of recent change in land use and fragmentation, and its indirect effect on niche competition in the ecosystem. In particular, the lack of canopy trees in fragmented forests caused by human land use decreases *D. malabaricum* seedling survival especially in the summer season. Moreover, predators of seeds of *D. malabaricum* have frequently been observed lately (G. Ravikanth, pers. obs.). It therefore seems that anthropogenic changes create a cascade of events at the local scale, which in turn could affect wide-scale genetic patterns. In light of this, it is important to evaluate genetic diversity at the species level and the historical connectivity among populations. Regarding the genetic connectivity and forest fragmentation, *D. malabaricum* is insect pollinated, and seeds are dispersed mainly via the Malabar grey hornbill (*Ocyceros griseus*) and occasionally by other large birds such as imperial pigeons and wood pigeons (Ganesh and Davidar 2001; Shivanna et al. 2003).

A study on fine-scale spatial genetic structure (SGS) and paternity analysis by Ismail et al. (2012) in the Coorg district revealed that the majority of pollination events occurred within sacred groves and did not go beyond 290 m. Furthermore, the proportion of short-distance mating events (<100 m) was much larger in forest patches with low *D. malabaricum* density than in stands with larger population sizes. Although no difference in genetic diversity could be detected among life stages (adults, saplings, seedlings, and embryos), there was a significant increase in relatedness among juveniles within the shortest distance class (<100 m). This was interpreted as the first signs of increased inbreeding due to fragmentation. Although gene flow between neighboring forest patches did occur, and the maximum distance of pollination detected was 23.6 km, it is not known whether this is enough to maintain genetic connectivity and population genetic structure at a larger scale still needs to be analyzed.

In the present study, we used 11 species-specific nuclear SSR (nSSR; simple sequence repeat) markers (Hemmilä et al. 2010) and one chloroplast SSR (cpSSR, Weising and Gardner 1999) to investigate the genetic diversity and structure of *D. malabaricum* across its distribution range and discuss implications for conservation efforts in Western Ghats. More specifically we (1) compare population genetic structure at biparentally inherited nSSRs (dispersed through pollen and seeds) and maternally inherited cpSSR (dispersed only through seeds), (2) assess the impact of the Palghat and Shencottah gaps on genetic structure, (3) infer population demographic history by approximate Bayesian computation (ABC) approach and finally, (4) based on these results offer some suggestions for conservation and management strategies.

## Materials and Methods

### Study site

Leaf samples from 343 individuals were collected in July 2010 from twelve populations located at 8.5°N–14.9°N in the Western Ghats (Fig. 1) and categorized according to the tree's height and diameter at breast height (dbh). The samples include 167 adults (dbh more than 10 cm), 72 juveniles (height 1 m or above, but dbh <10 cm), and 104 seedlings (height <1 m, i.e., 1 or 2 years old). Adults were sampled in all populations, whereas juveniles were sampled in five populations and seedlings in seven. In total, 8–51 individuals were sampled in each population (Table 1). Populations represented different degrees of disturbance, ranging from small and disturbed populations to large and protected ones. Sampling was performed in quadrats of size 10 × 10 m. The number of quadrats depended on the population size and was limited to 20 in large populations (>100 individuals). Leaves were collected from each individual and stored at −80°C prior to DNA extraction.

**Table 1:** Location, sample size, and values of genetic diversity parameters; gene diversity (*h*), allelic richness (*A*_[n]_), and fixation index (*F*_IS)_ for nuclear SSRs and cpSSR of 12 populations of *Dysoxylum malabaricum*

				Age class					Nuclear SSRs			cpSSR	
							
Populations	Disturbance^1^	Latitude (°*N*)	Longitude (°*E*)	Adults	Juveniles	Seedlings	Total		*h*	*A*_[14]_	*F*_IS_	*h*	*A*_[8]_
1. Yakambi	C	14.85	75.11	10	0	16	26		0.639	4.118	0.085	0.803	4.643
2. Jadegadde	C	14.80	74.74	6	0	16	22		0.621	4.339	0.205^1^	0.909	5.799
3. Hittalahalli	C	14.79	74.80	16	16	16	48		0.683	4.809	0.061	0.840	5.006
4. Navangere	B	14.56	74.94	19	11	8	38		0.595	4.388	0.176^1^	0.755	4.526
5. Tavanandi	A	14.46	74.80	19	14	17	50		0.594	4.260	0.073	0.855	5.167
6. Sarekoppa	C	14.44	74.51	10	16	16	42		0.579	4.088	0.140^1^	0.757	4.059
7. Agumbe	A	13.80	75.00	12	0	0	12		0.623	4.299	0.165	0.788	4.224
8. Coorg	B	12.40	75.90	15	15	15	45		0.706	5.253	0.089^1^	0.809	4.625
9. Peria	C	11.85	75.80	8	0	0	8		0.733	5.378	0.131	0.429	2.000
10. Sholayar	A	10.30	76.71	19	0	0	19		0.572	4.309	−0.042	0.696	3.242
11. Periyar Tiger Reserve	A	9.53	77.20	17	0	0	17		0.535	3.761	0.087	0.728	3.668
12. Arippa	A	8.50	76.98	16	0	0	16		0.636	4.642	0.033	0.642	2.996
			Total	167	72	104	343	Mean	0.626	4.470	0.074	0.751	4.163

1 Disturbance level is categorized as A, low disturbance and >50 individuals; B, medium disturbance; and C, high disturbance and low population size.

### DNA extraction and amplification

DNA was extracted using a modified CTAB protocol (Doyle and Doyle 1987). As the quality of the extracted DNA was not satisfactory, whole genome of each extracted DNA sample was amplified by using Illustra Genomiphi DNA amplification V2 kit (GE Healthcare Limited, Buckinghamshire, U.K.). Eleven species-specific nSSRs (locus Dysmal 1, 2, 3, 7, 9, 13, 14, 17, 18, 22, and 26; Hemmilä et al. 2010) and one cpSSR (cpSSR; locus ccmp7; Weising and Gardner 1999) were examined in this study. Initially, five “universal” cpSSR loci (ccmp 2, 4, 5, 7, and 10; Weising and Gardner 1999) were screened, but only one amplified in *D. malabaricum*. The primer pairs of the selected loci were mixed into four multiplex sets and amplified using Type-it Microsatellite PCR kit (Qiagen, Venlo, Netherlands) in 6.0 μL mixtures containing 1.2 μL of 1–10 ng of genomic DNA, 3.0 μL of Multiplex PCR master mix buffer, 1.2 μL of H_2_O, and 0.6 μL of primer mix (with the concentration of each primer pair adjusted to 1–2 μmol/L). Samples were amplified by a DNA thermal cycler (Takara Bio Inc., Shiga, Japan) using the following program: initiation of hot-start DNA polymerase and denaturation at 95°C for 15 min; 32 cycles of 95°C for 30 sec, 57°C for 30 sec, and 72°C for 30 sec; and a final 30 min extension step at 72°C. PCR products were loaded on a MegaBACE1000 (GE Healthcare Life Science) and genotyped using the fragment profiler v. 1.2 software (Amersham Biosciences, Buckinghamshire, U.K.). In cases where amplification and/or genotyping failed, the procedure was tried one more time to avoid missing data. Three genotype data sets were created; one for all individuals, one for adults only and one for seedlings and juveniles only. These three data sets were analyzed separately in order to allow comparisons between age classes. For this purpose, the data set containing adults was reduced to retain only the populations where juvenile and seedling data was also available.

### Data analysis

#### Nuclear SSR

##### Summary statistics

Null alleles arising from mutations in primer binding regions, or failure of amplification of longer fragments, may influence microsatellite genotyping. *F*-statistics may therefore be positively biased by false homozygotes. To check for the presence of null alleles, data were analyzed with FreeNA (Chapuis and Estoup 2007). FreeNA estimates unbiased *F*_ST_ in microsatellite data sets containing null alleles using the ENA (excluding null alleles) method, which detects unexpected homozygosity patterns. The genetic diversity parameters within each population were evaluated by determining the gene diversity (*h*; Nei 1987), allelic richness based on seven diploid individuals (*A*_[14]_; El Mousadik and Petit 1996) and the fixation index (*F*_IS_) using the computer program FSTAT 2.9.3 (hereafter, FSTAT, Goudet 1995, 2001). The significance of the deviation of *F*_IS_ values from 0 was estimated for each locus and across the loci for each population on the basis of 1000 randomizations using FSTAT. Genotypic disequilibrium was tested for all locus pairs in each population by randomization. The resulting *P*-values (=0.05) were adjusted applying a sequential Bonferroni correction (Rice 1989). To evaluate whether the populations examined here had experienced recent bottlenecks, we employed the BOTTLENECK 1.2.02 software (hereafter, BOTTLENECK analysis; Piry et al. 1999; Cornuet and Luikart 1996) under both the infinite allele mutation model (IAM) and the two-phase model (TPM; 30% of multistep mutation and 70% single-step mutation) assumptions.

##### Population structure

The degree of genetic differentiation among populations was evaluated by calculating the overall fixation index (*F*_ST_; Weir and Cockerham 1984) and its confidence intervals (95 and 99%), determined on the basis of 1000 bootstrapping replicates, using FSTAT. Pairwise *F*_ST_ values were also calculated, and the significance of the pairwise population differentiation was tested by randomizing multilocus genotypes between pairs of populations using FSTAT. We also calculated the standardized values of *G*_ST_, known as *G’*_ST_ (Hedrick 2005), which ranges from 0 to 1. Isolation by distance (IBD; Wright 1943) was evaluated with GenAlEx 6.4 (Peakall and Smouse 2006) using Rousset's (1997) method, which tests for statistical association between pairwise population differentiation (*F*_ST_/(1 − *F*_ST_)) and the natural logarithms of direct minimum geographic distance among populations. The genetic relationships among populations were evaluated by generating a Neighbor-joining (NJ) tree based on the *D*_A_ genetic distances (Nei et al. 1983), using Populations 1.2.30 BETA software (Langella 2007). The statistical confidence in the topology of the tree was evaluated by 1000 bootstraps derived using the same software. The NJ tree was reconstructed on a topographic map using Mapmaker and GenGIS2 softwares (Parks et al. 2009).

For inferences on population structure, the software STRUCTURE was used (Pritchard et al. 2000; Hubisz et al. 2009). It performs Bayesian assignment of individuals to a given number of genetic clusters (*K*), assuming that each cluster is in Hardy–Weinberg and linkage equilibria. Here, *K* = 1 through *K* = 15 were investigated under the correlated allele frequencies model by running 100,000 iterations of each *K*, with a burn-in length of 100,000 iterations, and averaging the results over 20 runs. Data on sampling location was used in the LOCPRIOR function, which can further assist the clustering. To help determine the optimal *K*, *ΔK* was calculated as described by Evanno et al. (2005). The distributions of probability of the data (LnP(D)) and the *ΔK* values were visualized in the STRUCTURE HARVESTER software (Earl and vonHoldt 2012). Bar charts for the proportions of the membership coefficient of each individual in STRUCTURE analysis over 20 runs for each *K* were summarized using CLUMPP (Jakobsson and Rosenberg 2007) and visualized in DISTRUCT (Rosenberg 2004).

#### Chloroplast SSR

##### Summary statistics

The haplotype frequencies over populations were visualized on a map using the “Pies on map” function in Genetic Studio (Dyer 2009). The gene diversity (*h*), haplotype richness based on eight haploid individuals (*H*_[8]_; El Mousadik and Petit 1996) were calculated using the CONTRIB software (developed by RJ Petit; http://www.pierroton.inra.fr/genetics/labo/Software/Contrib/).

##### Population structure

The population differentiation measurement, *R*_ST_ (Slatkin 1995; Pons and Petit 1996), which takes into account the genetic distance (i.e., number of repeat differences) between ordered haplotypes, was calculated. To test whether *R*_ST_ values were significantly higher than the values of unordered haplotype-based *G*_ST_, 1000 permutations were evaluated in the software Permut & cpSSR (developed by RJ Petit). Pairwise *R*_ST_ values were calculated in Arlequin 3.1 and IBD was evaluated with the two matrices of *R*_ST_/(1 − *R*_ST_) and the natural logarithms of geographical distance in GenAlEx. The standardized values of *G’*_ST_ were also calculated. An NJ tree based on the (*δμ*)^2^ genetic distances (Goldstein et al. 1995) was constructed using the software Populations (Langella 2007) and modified in the GenGIS2 software, as done on nSSR data.

#### Nuclear and chloroplast SSR

##### Comparison of individual-based SGS between nSSRs and cpSSR

As IBD was significant for both genomes (Fig. S1), individual-based SGS was examined. To compare the SGS in the nuclear and cp genomes in detail, a spatial autocorrelation analysis was performed separately for the nSSR and cpSSR data sets. For nSSR data, multilocus genotypic distances between individuals were calculated according to Peakall et al. (1995) and then spatial autocorrelation coefficients, *r* (Smouse and Peakall 1999), were calculated for each distance class of 100 km using GenAlEx. Similarly, squared values of repeat number among haplotypes were considered as genetic distance and the spatial autocorrelation was analyzed for cpSSR data. The upper and lower 95% confidence intervals around *r* were determined with 999 bootstraps, and the statistical significance of the autocorrelation was tested with 999 permutations. Furthermore, the heterogeneity in SGS among genomes was tested with the single-distance class (*t*^2^) and multidistance class (ω) criteria by the method of Smouse et al. (2008) implemented in GenAlEx.

##### Demographic history

**Figure 2:**
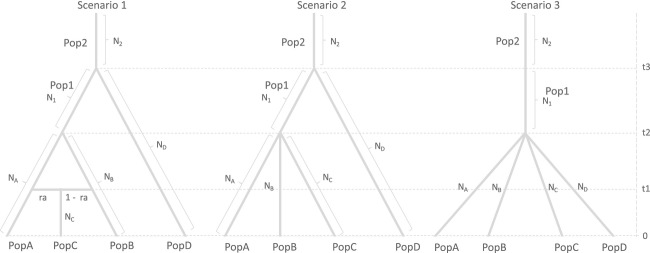
The three scenarios of population demography examined in DIYABC. t# is time scale measured in generations and N# is effective population size of the corresponding populations (Pop A, B, C, D, 1, and 2) during the time period (e.g., 0–*t*1, *t*1–*t*2, *t*2–*t*3).

Recently, ABC has emerged as a powerful and flexible approach to estimate demographic and historical parameters and to quantitatively compare alternative scenarios (Bertorelle et al. 2010). The software DIYABC v1.0.4.39 (Cornuet et al. 2008) was used to infer past demography. Four populations were defined based on the results from the STRUCTURE analysis (Fig. 5) and the NJ tree (Fig. S2); PopA (population 1–3), PopB (population 4–6), PopC (population 7, Agumbe), and PopD (population 8–12). We examined three simple demographic scenarios (Fig. 2). In scenario 1 PopC (Agumbe population), which appeared to be admixed in the STRUCTURE analysis, is assumed to have originated from the admixture of PopA and PopB at time *t*1. The rate of admixture from PopA to PopC was set as “ra” and the one from PopB to PopC was set as “1 - ra”. At time *t*2, PopB merged with PopA and the southern PopD merged with PopA and PopB at *t*3. In scenario 2 PopB and PopC were merged with PopA at time *t*2, and PopD was merged with PopA at *t*3. Finally scenario 3 corresponds to a simple population split scenario where PopA, PopB, PopC, and PopD all merged simultaneously at time *t*2. A population size change of the ancestral population (Pop1 and Pop2) was assumed at *t*3 in each scenario. Importantly, the models assume that there is no migration among populations under any of the three scenarios.

As DIYABC requires that one population traces back as the ancestral population, PopA was chosen in all scenarios, although the level of genetic diversity at SSR loci was almost similar in all populations. This arbitrary choice had no impact on the results, as additional analyses using different ancestral populations produced consistent results. The change in number of repeats followed a generalized stepwise mutation model (GSM; Estoup et al. 2002) and single nucleotide indels (SNI) were also allowed. The mutation rate of the former was assumed to be higher than the mutation rate of the latter. The default values of the priors were used for all parameters (Table S1). The mean values of the expected heterozygosity (*H*_E_) and the number of alleles (*A*) were used as summary statistics for each of the three populations, and for population pairs, *F*_ST_ was also used. One million simulations were performed for each scenario, and the most likely scenario was evaluated by comparing posterior probabilities with the logistic regression method. The goodness-of-fit of the three scenarios were also assessed by a principal component analysis (PCA) using the option “model checking” in DIYABC.

## Results

### Nuclear SSRs

Genotyping of nSSRs was successful in 86% of the cases and only three individuals did not amplify at any locus. Missing data was mainly confined to the two seedling groups of Navangere and Hittalahalli, where the success rate was only 14% and 19%, respectively, probably because of difficulties to keep the raw materials in good condition in the field and extract purified DNA from the samples of seedlings. As there was no significant difference between the three age classes in our initial analysis, we pooled the different age classes in each location, leading to a dataset with twelve populations.

### Summary statistics

**Figure 3:**
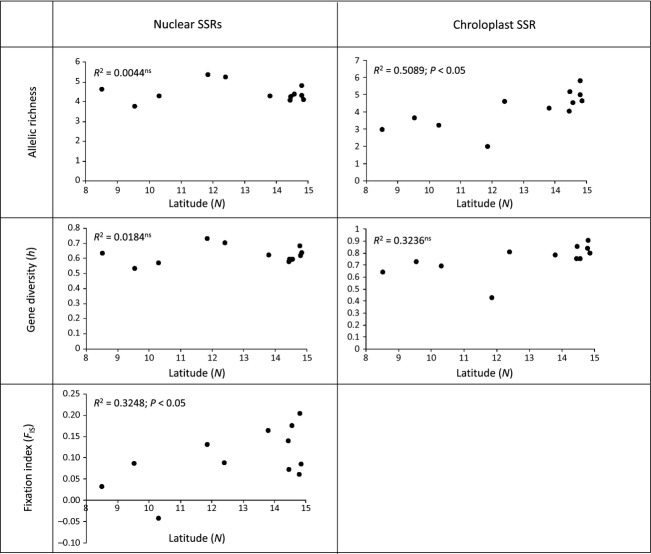
Relationship between genetic diversity parameters (allelic richness, gene diversity, and fixation index) and latitude, and longitude for 12 *Dysoxylum malabaricum* populations in nuclear and chloroplast SSRs.

As only a small amount of null alleles was detected and the *F*_ST_ values corrected for null alleles were almost the same as the uncorrected ones at all loci (Table S2), we used the original genotype data. Gene diversity and allelic richness were roughly similar between populations and were not significantly correlated with latitude (Table 1 and Fig. 3). The fixation index (*F*_IS_) value did not deviate significantly from zero at any locus in any population. However, significant positive overall values (*P* < 0.05) were detected in Jadegadde (2), Navangere (4), Sarekoppa (6) and Coorg (8) populations when the overall values were calculated. The *F*_IS_ was higher in the northern populations and the correlation with latitude was significant (*P* < 0.05). Genotype disequilibrium was significant at only one out of the 55 locus pairs (*P* < 0.05). In the BOTTLENECK analysis, a significant *H*_E_ excess (*P* < 0.05) was detected in the northernmost populations, Yakambi (1) population under the IAM, while significant *H*_E_ deficits were detected in Navangere (4) and Sholayar (10) populations under the TPM (*P* < 0.05).

### Population structure

**Figure 4:**
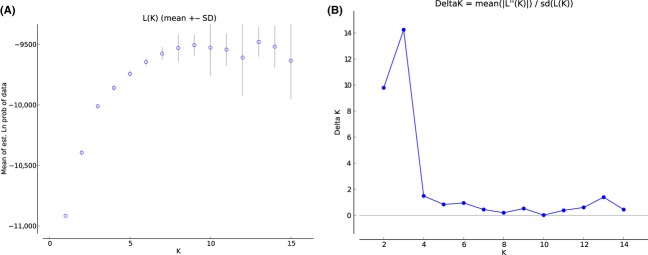
(A) The mean values of LnP(D) and standard deviation from 20 runs for each value of *K* = 1–12, (B) The distributions of *ΔK* over *K* = 1–15.

**Figure 5:**
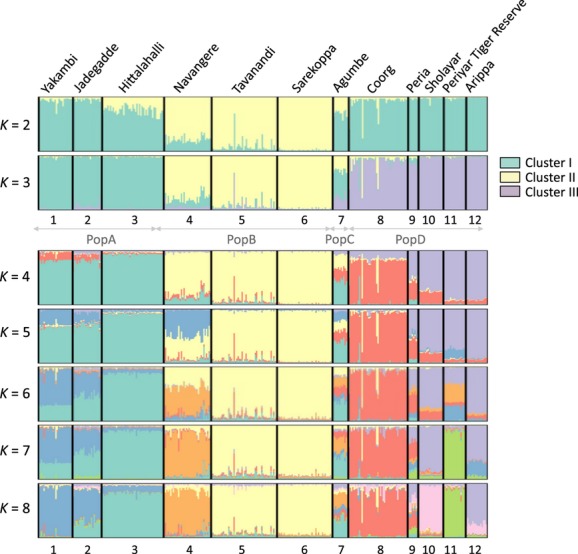
The proportion of the membership coefficient for each individual in 12 *Dysoxylum malabaricum* populations for the inferred clusters in *K* = 2–8 by the STRUCTURE analysis. The definitions of the four populations used in the DIYABC (PopA–D) are also shown.

The population differentiation indices, *F*_ST_ and *G*’_ST_, were 0.09 and 0.33, respectively. Although IBD was clear (Fig. S1), the variance in *F*_ST_ between closely located populations remains high, suggesting that there are several combinations of neighboring populations with strong genetic differentiation. In the STRUCTURE analysis, the probability of the data (LnP(D)) increased progressively up to *K* = 8 where it started to plateau (Fig. 4A). Indeed, several single populations (e.g., populations 3, 8, 10, 11, and 12) were assigned to specific clusters for higher values of *K* (Fig. 5). However, the clustering pattern for values of *K* > 4 showed complicated multimodality, that is, the assignment of individuals to clusters is inconsistent between runs, which indicates that such models are difficult to fit to the data. On the other hand, Δ*K* indicated that the optimal number of *K* was three (Fig. 4B). We therefore restricted further analyses to *K* = 3 (Fig. 5). For *K* = 3, the five southernmost populations, distributed over more than half of the species distribution range, form a separate cluster. The northernmost populations divide into two clusters. The overall pattern corresponds well with a latitudinal gradient of populations. The Agumbe population was not fully assigned to any of the three clusters; rather, it appears to be admixed (Fig. 5). Although several nodes were poorly supported by bootstraps, the result of the NJ tree showed a similar pattern as the STRUCTURE analysis (Fig. S2). Contrary to our expectations, the two geographic gaps did not have a clear effect on population genetic structure. Instead, a genetic gap was detected north of the two geographic gaps.

### Chloroplast SSR

#### Summary statistics

A total of twenty-four alleles were detected in ccmp7 (Fig. 1 and Table S3). All individuals were successfully amplified at this locus. The allelic richness showed a significant correlation with latitude, with highest values in the north (Fig. 3 and Table 1). The same trend was observed for gene diversity, but the cline was not significant. The distribution of haplotypes was well ordered with allele length increasing from north to south.

#### Population structure

The haplotype distribution pattern was mirrored in the NJ tree showing two northern and southern groups (Fig. S2). Significant IBD was found (Fig. S1) and also, alleles seemed ordered in one northern and one southern lineage (Fig. 1). Accordingly, *R*_ST_ was significantly higher than *G*_ST_ (0.689 and 0.203, respectively, *P* < 0.01) indicating the presence of a phylogeographic structure. The level of population differentiation obtained with *G*’_ST_ was 0.87, which is higher than the value detected with nSSRs (*G*’_ST_ = 0.33). Overall, the genetic structure in the cpSSR was clearly different from the one detected in nSSRs, but was in line with the nSSR in that there was no relationship with the two geographical gaps (Figs. 1 and S2).

### Nuclear and chloroplast SSRs

#### Comparison of individual-based SGS between nSSRs and cpSSR

**Figure 6:**
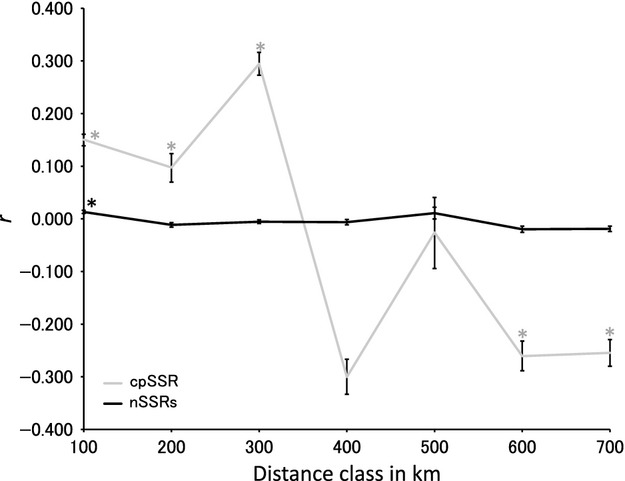
Spatial genetic structure in nuclear and chloroplast SSRs evaluated by the autocorrelation coefficient, *r* (Smouse and Peakall 1999). The asterisk marks the distance classes showing a significant coefficient.

The SGS pattern was different between the genomes and the values of spatial autocorrelation coefficients and *r* were positively significant from the first to the third distance classes in the cpSSR while it was significant only in the first distance class for nSSR. Significantly negative values were reached in the sixth and seventh distance class for the cpSSR. The heterogeneity tests showed that the entire SGS was different between the two genomes (ω = 42.120; *P* = 0.001, Fig. 6). This indicates that the nuclear genome is more locally structured than the cp one, as spatial autocorrelation was found over larger distances in the cp marker.

#### Demographic history

Scenario 3, which corresponds to a simple simultaneous split of the four populations had by far the highest posterior probability (0.6146, 95%, CI = 0.5596–0.6697) (Table 2). The median values of the effective population size were 9230, 6220, 5610, 9040, 6770, and 4810, for N_A_ (PopA), N_B_ (PopB), N_C_ (PopC), N_D_ (PopD), N_1_ (Pop1) and the ancestral N_2_ (Pop2), respectively, (Table S4). The median values of the divergence time, *t*2 and the time of population size change, *t*3 were 1760 and 4340 generations ago, respectively. If we assume a generation time of 25 years (Ravikanth et al. 2008, G. Ravikanth, pers. obs.), the divergence time of the three populations would be 44,000 years ago and the time of the ancestral population size change 108,500 years ago. However, the posterior distribution pattern suggested that *t*3 is poorly estimated (Fig. S3). The median value of the mutation rate of SSR and SNI at examined loci were estimated at 5.09 × 10^−4^ and 2.38 × 10^−5^, respectively. Observed values of the expected *H*_E_, the number of alleles (*A*) in each population and *H*_*E*_, *A* and *F*_ST_ for all possible combinations of population pairs did not differ significantly from simulated values based on parameters values drawn from the posterior distributions for scenario 3. In the PCA, the observed data (large yellow dot) is among the values obtained from the posterior distribution (large blue dots), the small dots corresponding to the prior distribution. This indicates a good fit of the posterior distribution based on scenario 3 to the data (Fig. S4).

**Table 2:** Posterior probability of each scenario and its 95% confidence interval based on the logistic estimate by DIYABC

Scenario	Posterior probability	95% CI (lower–upper)
1	0.2244	0.1779–0.2709
2	0.1609	0.1314–0.1905
3	0.6146	0.5596–0.6697

## Discussion

### Genetic diversity of *D. malabaricum*

In the present study we investigated genetic diversity and population structure across the range of *D. malabaricum* in the Western Ghats using both nuclear and cpSSRs. Firstly, as regeneration was poor, potentially because of overharvesting or newly generated niche competition induced by human land use, we aimed to compare genetic diversity among age classes, expecting lower genetic diversity in younger individuals. However, no difference was found between them. This pattern was also detected in a fine-scale genetic study of *D. malabaricum* in the southern part of the Western Ghats (Ismail et al. 2012). Indeed, as trees are typically harvested after about 80 years, old adult trees are still relatively frequent (Y. Tsuda and G. Ravikanth, field obs.). Thus, these older trees would still contribute to the reproductive success and help maintain the genetic diversity in younger cohorts. In addition, as clusters of populations were detected by the STRUCTURE analysis, sufficient gene flow between populations within clusters may occur, and it would also contribute to the maintenance of genetic diversity in individual populations. Although gene diversity and allelic richness did not show geographic patterns in nSSRs, the *F*_IS_ values were significantly higher in the northern populations than in the southern populations, suggesting that inbreeding is higher in the north. This pattern gives support to the classification of populations according to their perceived level of disturbance as populations classified as highly disturbed (category C) are concentrated to the north, whereas most of the least disturbed populations (category A) are found in the south (Table 1). Even in the southern part of the Western Ghats (around the Coorg population which showed significant deviation of *F*_IS_ from 0 in this study), a recent study on parentage and kinship analysis of *D. malabaricum* in a fragmented agro-forestry landscape (Ismail et al. 2012) found that pollen dispersal between sacred groves prevented the build-up of inbreeding at different life stages (adults, sapling, seedlings, and embryos). Data from both southern and northern populations hence suggest that the higher values of *F*_IS_ in the northern populations detected in this study could indeed be due to inbreeding induced by small population sizes and relative isolation at the margin of the species together with serious disturbance. This interpretation is further supported by the detection of a bottleneck in the northern most population, Yakambi (1) and by the observation of a smaller number of adult trees in this population (<20 adult trees) than in other northern marginal populations (e.g., Hittalahalli, Navangere, and Sarekoppa) (>60 individuals, Y. Tsuda and G. Ravikanth, field obs.).

### Population structure and demographic history

Three and two genetic clusters were observed with nSSRs and cpSSR, respectively, but notably, the Palghat and Shenocottah gaps did not appear to be reproductive barriers for either genome. The distributions of the clusters were not congruent, for nuclear and cp markers and an explanation could be that population demographic history differs between the genomes, reflecting differences in inheritance and dispersal modes. The cpSSR revealed a clear geographical gradient in distribution of ordered haplotypes, which is likely informative although only one locus was examined.

Genetic clusters in the nuclear genome appear well defined over all populations except Agumbe (7), in which an admixture-like pattern was detected by the STRUCTURE analysis. However, the ABC analysis suggested that the most likely scenario was a simple population split into four population groups. The divergence time of the four groups was estimated to 1760 generations ago, or 44,000 years ago when we assume a generation time of 25 years for *D. malabaricum*. Estimating generation time remains difficult in species with long reproductive spans like forest trees (Petit and Hampe 2006). While *D. malabaricum* bears first fruits at 12–15 years of age it only reaches the canopy after around 25 years at which stage the trees acquire their full reproductive potential. If we assume that the generation time is around 25 years then the divergence time predates the last glacial maximum (LGM, 26,500–19,000 years before present) (Clark et al. 2009). During the LGM the Western Ghats shifted toward colder and drier climate than today, severely affecting the distribution of rainforests (Farooqui et al. 2010). In India, rainforests spread from isolated pockets only 4000–7000 years ago (Farooqui et al. 2010). Thus, these facts together with the results of the ABC analysis suggested that the divergence of *D. malabaricum* occurred before the LGM, and that refugia were formed in several places. Our results from the STRUCTURE and ABC analysis support a model where modern populations of *D. malabaricum* were founded from one refugium in the south and two or three refugia in the north. Indeed, one such rainforest refugium has been found in southern Western Ghats in a palynological study (Farooqui et al. 2010). Also, although *D. malabaricum* has a clinal distribution, important environmental variables such as mean annual rainfall and length of dry season, remain largely constant throughout the range (Prasad et al. 2009). In conclusion, the overall population genetic structure was therefore likely formed by historical gene flow and past climatic events rather than recent human activities and adaptation to local climate.

The finding that an instant split into four groups fit the data better than a model with three groups, where two of them merged to found a fourth, admixed population is in itself interesting. This suggests that the Agumbe population is a unit of its own, rather than an admixed population. This interpretation is supported by a recent study where ancestral polymorphism was explicitly taken into account in the case of freshwater fish (Sousa et al. 2012). In this study ABC analysis was performed on simulated and empirical data, and it was found that the genetic structure was better explained by a population split model without admixture (a similar model to scenario 3 in the present study) than by a model with admixture, even when STRUCTURE analysis showed an admixture-like pattern. In fact, one of the issues with inferences of population demography is that ancestral shared polymorphisms are often difficult to separate from admixture or gene flow (Sousa et al. 2012), and are extremely common in trees (e.g., Chen et al. 2010). Thus, the ABC approach in the present study provides demographic information for the conservation of relevant units in the target species. However, there are some caveats. As pointed out earlier the ABC analysis implemented in DIYABC assumes no gene flow among populations. The rather low level of population differentiation (overall *G*’_ST_ = 0.33) suggests that there may be some gene flow, although the clear STRUCTURE results suggest that recent gene flow may not be too important. Therefore, estimates of divergence time and effective population sizes will likely be biased downwards and upwards, respectively. However, the main results of pre-LGM divergence would not be changed even when gene flow is taken into account as ignoring gene flow would lead to underestimating the divergence time.

Additional insights on the demographic history of *D. malabaricum* can be gained by comparing nSSRs and cpSSR. Since *D. malabaricum* is insect pollinated and seeds are dispersed via birds, one would expect more genetic structure in the nuclear genome than in the cp one (Petit et al. 2005), which was indeed found. The Coorg (8) population primarily belonged to the southern cluster in the STRUCTURE analysis, while it was assigned to the northern group based on cpSSR variation. This suggests that organelle capture occurred between the northern and the southern lineages in the middle of the species range by repeated hybridization and backcross events among them through pollen flow from the southern lineage. However, it should be noted that we cannot rule out the possibility that the contrasting patterns could stem from a difference in mutation rates between the cp and nuclear genomes.

Although there was some discrepancy in geographic distributions of genetic groups between nSSRs and cpSSRs groups, both genomes showed clear clusters, suggesting reproductive barriers in the species distribution range and historically limited gene flow among them. However, the Palghat and Shencottah gaps did not constitute such barriers. Regarding the seed flow, although the feeding behavior of hornbills tends to cause aggregation of individuals, they have been observed to fly over unsuitable habitats between fragments and sometimes even migrate several hundred kilometers (Raman and Mudappa 2003). This ability is apparently sufficient to maintain high level of genetic connectivity between populations within the northern and southern cpSSR groups of *D. malabaricum*. Therefore, it may not come as a surprise that the 40-kilometer wide Palghat gap did not constitute a barrier to gene flow. On the other hand, while the longest pollen dispersal distance was 23.6 km, the mean pollen dispersal was 1205 m in high-density stands and 600 m in low-density stands, via beetles and thrips (Ismail et al. 2012). Thus, pollen dispersal in this species appears more restricted than seed dispersal. The results of the SGS analysis supported this and showed wider significant spatial autocorrelation in the cpSSR than in nSSRs. Moreover, in spite of multimodalities in the STRUCTURE analysis, clear clustering was also found and several single populations were assigned to specific clusters for number of *K* as high as *K* = 8 and this genetic differentiation at a local scale also suggested limited pollen flow among populations.

### Implications for conservation

The geographic patterns of genetic diversity of *D. malabaricum* that was found appears to reflect the species’ natural population history rather than recent human impact. However, although clear loss of genetic diversity by human activities was not suggested by this study, ongoing activities (e.g., harvesting and deforestation) and its secondary impact (niche competition in the ecosystem) as reflected in the imbalance between age classes are still serious concerns for the long-time survival of the species. Especially, evidence of inbreeding in the northern populations, which are also among the most disturbed ones, and of a recent bottleneck in the Yakambi population, may be the first signals of unsuccessful regeneration due to human activities. Our study hence suggests that conservation priority should be given to these northern populations and it is recommended to start efforts to evaluate regeneration dynamics in this region. This would entail fine-scale analysis of genetic structure among age classes. Our data also suggest the presence of long distance seed dispersal at least within the two groups detected with cpSSR. Given the importance of long-distance dispersal in *D. malabaricum*, it is desirable to maintain populations of its seed disperser, the Malabar grey hornbill. Luckily, this species has recently been listed by the IUCN as “Least concern” (BirdLife International 2012). However, as discussed above, the distributions of haplotypes were largely different between the northern and southern groups. Therefore, more populations would be needed between the two groups to evaluate the distribution of the genetic barriers of *D. malabaricum*.

Wide-scale genetic structure studies have provided useful information on conservation units and seed zones and guidelines for restoration or plantation efforts in forest conservation and tree breeding programs of many tree species, especially economically important ones (Bucci and Vendramin 2000; Lefèvre 2004; Tsuda and Ide 2005; Sutherland et al. 2010). This information provided by wide-scale genetic structure could also be relevant to the conservation of *D. malabaricum*. Based on the present study, we suggest that *D. malabaricum* should be managed as four separate units, according to the clusters found with nSSRs. Although the population structure of cpSSR was not congruent with this, priority should be given to the results of the nSSRs for two reasons; the inference is based on a larger number of independent loci, and it revealed more fine-scale diversity which might be lost if the species were to be managed as only two units. Therefore, the minimum ambition should be to conserve one population each from the four clusters as a representative for their respective genetic diversity. The status of the Agumbe population as a separate unit is not fully settled but should it be an admixed population, it still harbors genetic diversity from all the three other known lineages and thus specific conservation efforts would be justified. Although currently large-scale transplantation or restoration of individuals may not be needed in order to mitigate inbreeding over most of the species’ range, the proposed conservation units would be informative for adaptive management of this natural resources. Moreover, it is required to evaluate the ecological dynamics of not only the target species but also the whole ecosystem associated to it and to design a more practical approach of ecosystem management in this biodiversity hotspot.

## Data Accessibility

DIYABC input file and genotype data have been uploaded to DRYAD doi: 10.5061/dryad.g2b10.

Prior distributions of parameters used in DIYABC and distribution of cp haplotypes over the twelve populations and have been uploaded online as supporting information.

## Supporting Information

**Figure S1.** The relationships between the matrix of pairwise differentiation described as *F*_ST_/(1 − *F*_ST_) for nuclear SSRs (A) and *R*_ST_/(1 − *R*_ST_) for chloroplast SSR (B), and the matrix of the natural logarithm of geographic distance (in km) in the 12 populations.

**Figure S2.** The population-based neighbor-joining tree in nuclear SSRs (A, *D*_A_ distance, Nei et al. 1983) and chloroplast SSR (B, (*δ*μ)^2^ distance, Goldstein et al. 1995). The bootstrap values were shown when the value exceeds 50% in (A).

**Figure S3.** The prior and posterior distributions for each parameter obtained by DIYABC.

**Figure S4.** The principal component analysis (PCA) obtained by DIYABC.

**Table S1.** Prior distributions of the parameters used in DIYABC

**Table S2.** *F*_ST_ values per locus with and without correction for null alleles.

**Table S3.** Distribution of chloroplast haplotypes (alleles in ccmp7) in each of the 12 populations of *Dysoxylum malabaricum*.

**Table S4.** Demographic parameters obtained by DIYABC.

## References

[b1] Abdullah SA, Nakagoshi N (2007). Forest fragmentation and its correlation to human land use change in the state of Selangor, peninsular Malaysia. For. Ecol. Manage.

[b2] Aguilar R, Ashworth L, Galetto L, Aizen MA (2006). Plant reproductive susceptibility to habitat fragmentation: review and synthesis through a meta-analysis. Ecol. Lett.

[b3] Bacles CFE, Lowe AJ, Ennos RA (2006). Effective seed dispersal across a fragmented landscape. Science.

[b4] Bahulikar RA, Lagu MD, Kulkarni BG, Pandit SS, Suresh HS, Rao MKV (2004). Genetic diversity among spatially isolated populations of *Euryanitida* Korth. (Theaceae) based on inter-simple sequence repeats. Curr. Sci.

[b6] Bertorelle G, Benazzo A, Mona S (2010). ABC as a flexible framework to estimate demography over space and time: some cons, many pros. Mol. Ecol.

[b7] BirdLife International (2012). Ocyceros griseus. IUCN 2012. IUCN red list of threatened species.

[b8] Bucci G, Vendramin GG (2000). Delineation of genetic zones in the European Norway spruce natural range: preliminary evidence. Mol. Ecol.

[b9] Chapuis MP, Estoup A (2007). Microsatellite null alleles and estimation of population differentiation. Mol. Biol. Evol.

[b10] Chen J, Källman T, Gyllenstrand N, Lascoux M (2010). New insights on the speciation history and nucleotide diversity of three boreal spruce species and a Tertiary relict. Heredity.

[b11] Clark PU, Dyke AS, Shakun JD, Carlson AE, Clark J, Wohlfarth B (2009). The Last Glacial Maximum. Science.

[b12] Cornuet JM, Luikart G (1996). Power analysis of two tests for detecting recent population bottlenecks from allele frequency data. Genetics.

[b13] Cornuet JM, Santos F, Beaumont MA, Robert CP, Marin JM, Balding DJ (2008). Inferring population history with DIYABC: a user-friendly approach to Approximate Bayesian Computations. Bioinformatics.

[b14] Crandall KA, Bininda-Emonds ORP, Mace GM, Wayne RK (2000). Considering evolutionary processes in conservation biology. Trends Ecol. Evol.

[b15] D'Cruz E, Nair PKR, Prasannakumar V (2000). Palghat Gap - A Dextral Shear Zone from the South Indian Granulite Terrain. Gondwana Res.

[b16] Deshpande AU, Apte GS, Bahulikar RA, Lagu MD, Kulkarni BG, Suresh HS (2001). Genetic diversity across natural populations of three montane plant species from the Western Ghats, India revealed by intersimple sequence repeats. Mol. Ecol.

[b17] Doyle JJ, Doyle JS (1987). A rapid DNA isolation procedure for small quantities of fresh leaf tissue. Phytochem. Bull.

[b18] Dyer RJ (2009). GeneticStudio: a suite of programs for spatial analysis of genetic-marker data. Mol. Ecol. Resour.

[b19] Earl DA, vonHoldt BM (2012). STRUCTURE HARVESTER: a website and program for visualizing STRUCTURE output and implementing the Evanno method. Conserv. Genet. Resour.

[b20] El Mousadik A, Petit R (1996). High level of genetic differentiation for allelic richness among populations of the argan tree *Argania spinosa* (L.) Skeels endemic of Morocco. Theor. Appl. Genet.

[b21] Estoup A, Jarne P, Cornuet JM (2002). Homoplasy and mutation model at microsatellite loci and their consequences for population genetics analysis. Mol. Ecol.

[b22] Evanno G, Regnaut S, Goudet J (2005). Detecting the number of clusters of individuals using the software STRUCTURE: a simulation study. Mol. Ecol.

[b23] Farooqui A, Ray JG, Farooqui SA, Tiwari RK, Khan ZA (2010). Tropical rainforest vegetation, climate and sea level during the Pleistocene in Kerala, India. Quatern. Int.

[b24] Frankel OH (1974). Genetic conservation: our evolutionary responsibility. Genetics.

[b25] Ganesh T, Davidar P (2001). Dispersal modes of tree species in the wet forests of southern Western Ghats. Curr. Sci.

[b26] Goldstein DB, Ruiz Linares A, Cavalli-Sforza LL, Feldman MW (1995). Genetic absolute dating based on microsatellites and the origin of modern humans. Proc. Natl Acad. Sci. USA.

[b27] Goudet J (1995). Fstat version 1.2: a computer program to calculate F-statistics. J. Hered.

[b28] Goudet J (2001). http://www.unil.ch/izea/softwares/fstat.html.

[b29] Hamilton MB (1999). Tropical tree gene flow and seed dispersal. Nature.

[b30] Hedrick PW (2005). A standardized genetic differentiation measure. Evolution.

[b31] Hemmilä S, Mohana Kumara P, Gustafsson S, Sreejayan N, Raghavandra A, Vasudeva R (2010). Development of polymorphic microsatellite loci in the endangered tree species *Dysoxylum malabaricum* and cross amplification with *Dysoxylum binectariferum*. Mol. Ecol. Resour.

[b32] Hubisz MJ, Falush D, Stephens M, Pritchard JK (2009). Inferring weak population structure with the assistance of sample group information. Mol. Ecol. Resour.

[b33] Ismail SA, Ghazoul J, Ravikanth G, Uma Shaanker R, Kushalappa CG, Kettle CJ (2012). Does long distance pollen dispersal preclude inbreeding in tropical trees? Fragmentation genetics of *Dysoxylum malabaricum* in an agro-forest landscape. Mol. Ecol.

[b34] Jakobsson M, Rosenberg NA (2007). CLUMPP: a cluster matching and permutation program for dealing with label switching and multimodality in analysis of population structure. Bioinformatics.

[b35] Jha CS, Dutt CBS, Bawa KS (2000). Deforestation and land use changes in Western Ghats, India. Curr. Sci.

[b36] Jump AS, Peñuelas J (2006). Genetic effects of chronic habitat fragmentation in a wind-pollinated tree. Proc. Natl Acad. Sci. USA.

[b37] Khan MA (2007). Mapping of the genetic diversity of Dysoxylum malabaricum Bedd. A critically endangered and economically important tree species of the Western Ghats. M. Sc.

[b38] Kodandapani N, Cochrane MA, Sukumar R (2004). Conservation Threat of Increasing Fire Frequencies in the Western Ghats, India. Conserv. Biol.

[b39] Kumar AN (2009). Saving culture for biodiversity. Exploring the “Bio-Cultural” Heritage in conservation of 5 Rare, Endemic & Threatened (RET) Tree Species of Western Ghats of Kerala.

[b40] Langella O (2007). http://bioinformatics.org/~tryphon/populations/.

[b41] Lefèvre F (2004). Human impacts on forest genetic resources in the temperate zone: an updated review. For. Ecol. Manage.

[b42] Menon ARR, Balasubramanyan K (2006). Evaluation of plant diversity in unlogged and logged forest stands of varying intensities.

[b43] Menon S, Bawa KS (1997). Applications of Geographical Information Systems, remote sensing and a landscape ecology approach to biodiversity conservation in theWestern Ghats. Curr. Sci.

[b45] Moritz C (2002). Strategies to protect biological diversity and the evolutionary process that sustain it. Syst. Biol.

[b46] Murawski DA, Gunatilleke IAU, Bawa KS (1994). The effect of selective logging on inbreeding in *Shorea megistophyll* (Dipterocarpaceae) from Sri Lanka. Conserv. Biol.

[b47] Myers N, Mittermeier RA, Mittermeier CG, Kent GAB, da Fonseca J (2000). Biodiversity hotspots for conservation priorities. Nature.

[b48] Nageswara Rao M, Ganeshaiah KN, Uma Shaanker R (2001). Genetic diversity of Medicinal plant species in deciduous forest of South India: impact of harvesting and other anthropogenic pressures. J. Plant Biol.

[b49] Nageswara Rao M, Ganeshaiah KN, Uma Shaanker R (2007). Assessing threats and mapping sandal (*Santalum album* L.) resources in peninsular India: Identification of genetic hot-spot for *in-situ* conservation. Conserv. Genet.

[b50] Nair A, Gopalan SV, George S, Kumar KS, Shikano T, Merilä J (2012). Genetic variation and differentiation in Indirana beddomii frogs endemic to the Western Ghats biodiversity hotspot. Conserv. Genet.

[b51] Nathan SS, Kalaivani K, Sehoon K (2006). Effects of *Dysoxylum malabaricum* Bedd. (Meliaceae) extract on the malarial vector *Anopheles stephensi* Liston (Diptera: Culicidae). Bioresour. Technol.

[b52] Nei M (1987). Molecular evolutionary genetics.

[b53] Nei M, Tajima F, Tateno Y (1983). Accuracy of estimated phylogenic trees from molecular data. J. Mol. Evol.

[b54] Padmini S, Nageswara Rao M, Ganeshaiah KN, Uma Shaanker R (2001). Genetic diversity of *Phyllanthus emblica* in tropical forests of South India: impact of Anthropogenic Pressures. J. Trop. For. Sci.

[b55] Parks DH, Porter M, Churcher S, Wang S, Blouin C, Whalley J (2009). GenGIS: a geospatial information system for genomic data. Genome Res.

[b56] Peakall R, Smouse PE (2006). GENALEX 6: genetic analysis in Excel. Population genetic software for teaching and research. Mol. Ecol. Notes.

[b57] Peakall R, Smouse PE, Huff DR (1995). Evolutionary implications of allozyme and RAPD Variation in diploid populations of dioecious buffalo grass (*Buchloë dactyloides* (Nutt. (Engelm.). Mol. Ecol.

[b58] Petit RJ, Hampe A (2006). Some evolutionary consequences of being a tree. Annu. Rev. Ecol. Evol. Syst.

[b59] Petit RJ, Duminil J, Fineschi S, Hampe A, Salvini D, Vendramin GG (2005). Comparative organization of chloroplast, mitochondrial and nuclear diversity in plant populations. Mol. Ecol.

[b60] Piry S, Luikart G, Cornuet JM (1999). BOTTLENECK: a computer program for detecting recent reductions in the effective population size using allele frequency data. J. Hered.

[b61] Pons O, Petit RJ (1996). Measuring and testing genetic differentiation with ordered versus unordered alleles. Genetics.

[b62] Prasad V, Farooqui A, Tripathi SKM, Garg R, Thakur B (2009). Evidence of late palaeocene-early Eocene equatorial rain forest refugia in southern Western Ghats, India. J. Biosci.

[b63] Pritchard JK, Stephens M, Donnelly P (2000). Inference of population structure using multilocus genotype data. Genetics.

[b64] Raman TRS (2006). Effects of habitat structure and adjacent habitats on birds in tropical rainforest fragments and shaded plantations in the Western Ghats, India. Biodivers. Conserv.

[b65] Raman TRS, Mudappa D (2003). Correlates of hornbill distribution and abundance in rainforest fragments in the southern Western Ghats, India. Bird Conserv. Int.

[b66] Ramesha BT, Ravikanth G, Nageswara Rao M, Ganeshaiah KN, Uma Shaanker R (2007). Genetic structure of rattan, *Calamus thwaitesii* in core, and buffer and peripheral regions of three protected areas at central Western Ghats, India: do protected areas serve as refugia for genetic resources of economically important plants?. J. Genet.

[b67] Ravikanth G, Uma Shaanker R, Ganeshaiah KN (2000). Conservation Status of forests in India: a cause for worry?. J. Indian Inst. Sci.

[b68] Ravikanth G, Ganeshaiah KN, Uma Shaanker R, Uma Shaanker R, Ganeshaiah KN, Bawa KS (2001). Mapping genetic diversity of rattans in Central Western Ghats: identification of hot-spots of variability for *in situ* conservation. Forest genetic resources: status, threats and conservation strategies.

[b69] Ravikanth G, Rao N, Deepali Singh M, Chaluvaraju BS, Ganeshaiah KN, Uma Shaanker R (2008). Contrasting spatial patterns of distribution of genetic diversity in two important bamboo species in the central Western Ghats, India. J. Bamboo Rattan.

[b70] Ravikumar K, Ved DK (2000). 100 Red listed medicinal plants of conservation concern in Southern India.

[b71] Rawat VRS, Kishwan J (2008). Forest conservation-based, climate change-mitigation approach for India. Int. For. Rev.

[b72] Rice WR (1989). Analyzing tables of statistical tests. Evolution.

[b73] Robin VV, Sinha A, Ramakrishnan U (2010). Ancient Geographical Gaps and Paleo-Climate Shape the Phylogeography of an Endemic Bird in the Sky Islands of Southern India. PLoS ONE.

[b74] Rosenberg NA (2004). Distruct: a program for the graphical display of population structure. Mol. Ecol. Notes.

[b75] Rousset F (1997). Genetic differentiation and estimation of gene flow from F-statistics under isolation by distance. Genetics.

[b77] Shivanna KR, Gladwin J, Shaanker RU (2003). Reproductive ecology and population enrichment of endemic and critically endangered plant species of the Western Ghats.

[b78] Slatkin M (1995). A measure of population subdivision based on microsatellite allele frequencies. Genetics.

[b79] Smouse PE, Peakall R (1999). Spatial autocorrelation analysis of individual multi allele and multilocus genetic structure. Heredity.

[b80] Smouse P, Peakall R, Gonzales E (2008). A heterogeneity test for fine-scale genetic structure. Mol. Ecol.

[b81] Sork VL, Smouse PE (2006). Genetic analysis of landscape connectivity in tree populations. Landscape Ecol.

[b82] Sousa VC, Beaumont MA, Fernandes P, Coelfo MM, Chikhi L (2012). Population divergence with or without admixture: selecting models using an ABC approach. Heredity.

[b83] Sutherland BG, Belaj A, Nier S, Cottrell JE, Vaughan SP, Hubert J (2010). Molecular biodiversity and population structure in common ash (*Fraxinus excelsior* L.) in Britain: implications for conservation. Mol. Ecol.

[b84] Tsuda Y, Ide Y (2005). Wide-range analysis of genetic structure of *Betulamaximowicziana*, a long-lived pioneer tree species and noble hardwood in the cool temperate zone of Japan. Mol. Ecol.

[b85] Vidya TNC, Fernando P, Melnick DJ, Sukumar R (2005). Population differentiation within and among Asian elephant (*Elephas maximus*) populations in southern India. Heredity.

[b86] Weir B, Cockerham C (1984). Estimating F-Statistics for the Analysis of Population Structure. Evolution.

[b87] Weising K, Gardner RC (1999). A set of conserved PCR primers for the analysis of simple sequence repeat polymorphisms in chloroplast genomes of dicotyledonous angiosperms. Genome.

[b88] Whittaker RJ, Araújo MB, Jepson P, Ladle RJ, Watson JE, Willis KJ (2005). Conservation Biogeography: assessment and prospect. Divers. Distrib.

[b89] Wright S (1943). Isolation by Distance. Genetics.

