# Supplementary figures and images for: Genetic structure and demographic history of the endangered tree species Dysoxylum malabaricum (Meliaceae) in Western Ghats, India: implications for conservation in a biodiversity hotspot
Source: Ecol Evol. 2013 Aug 6;3(10):3233–48. doi: 10.1002/ece3.669 (PMC3797473; doi:10.1002/ece3.669)

Fig. S1

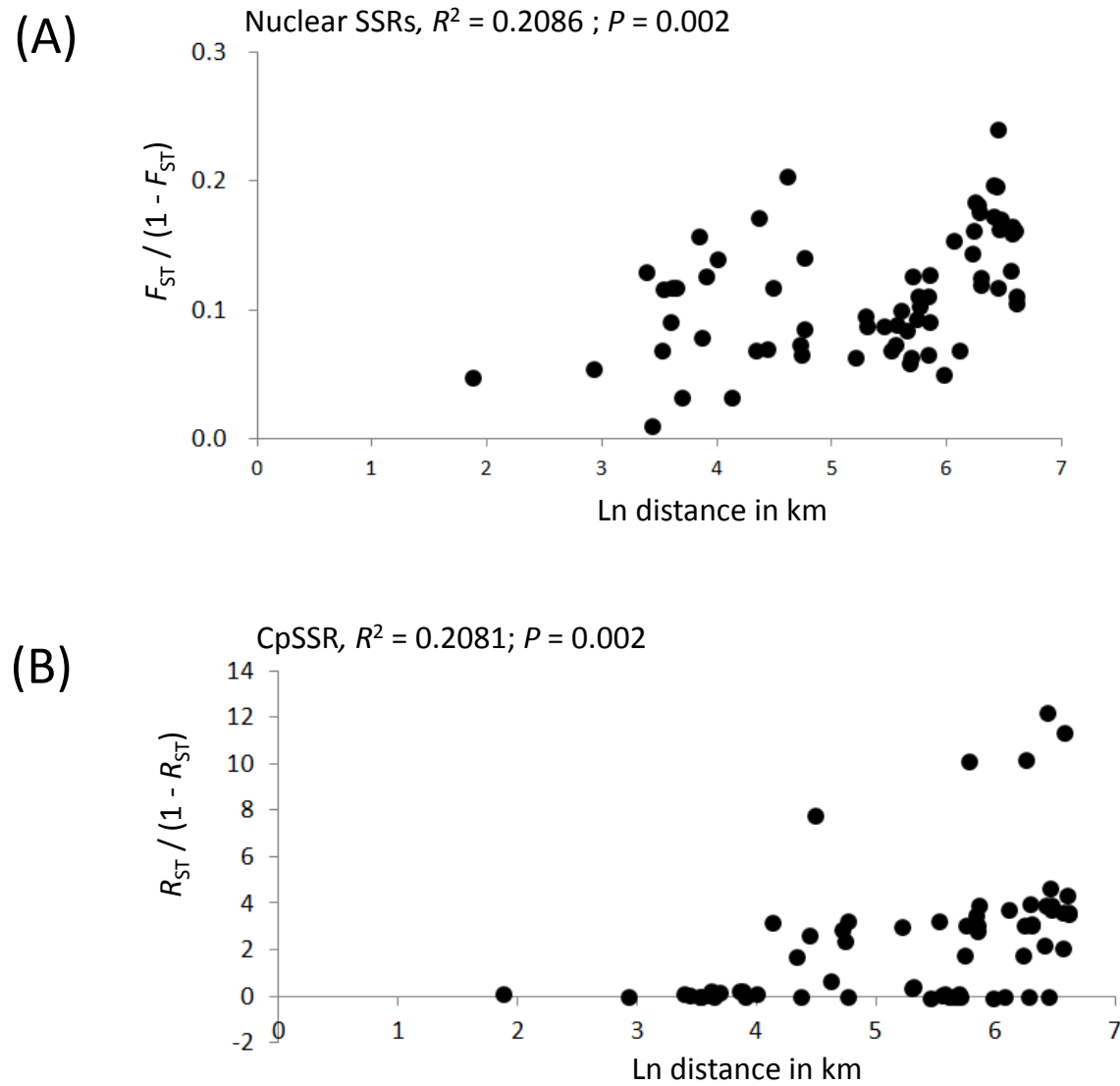

Supplement: Supplementary file 1 [file ece30003-3233-SD1.pdf]

Fig. S2

(A)

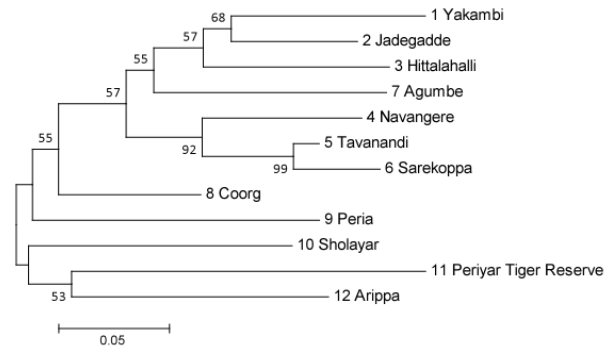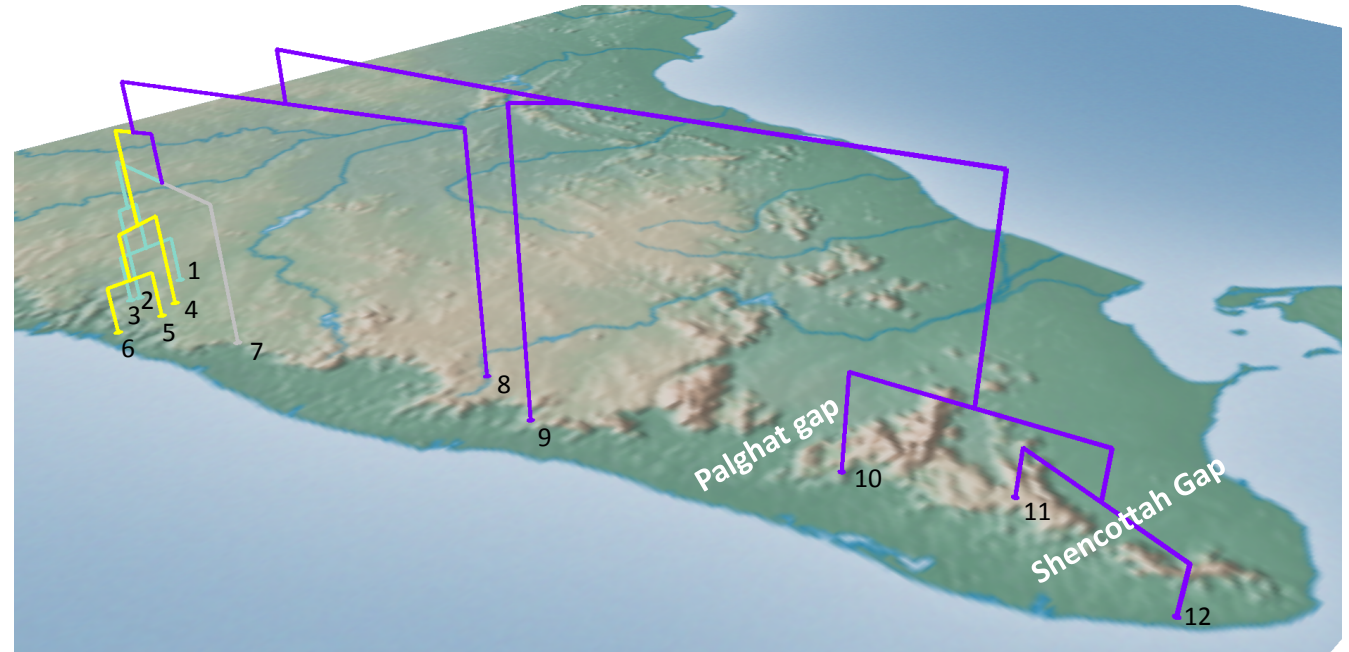

(B)

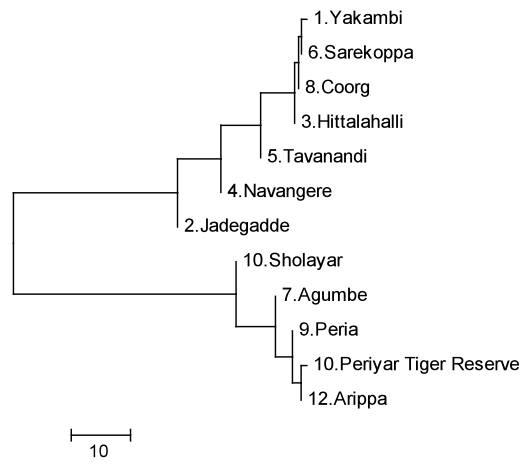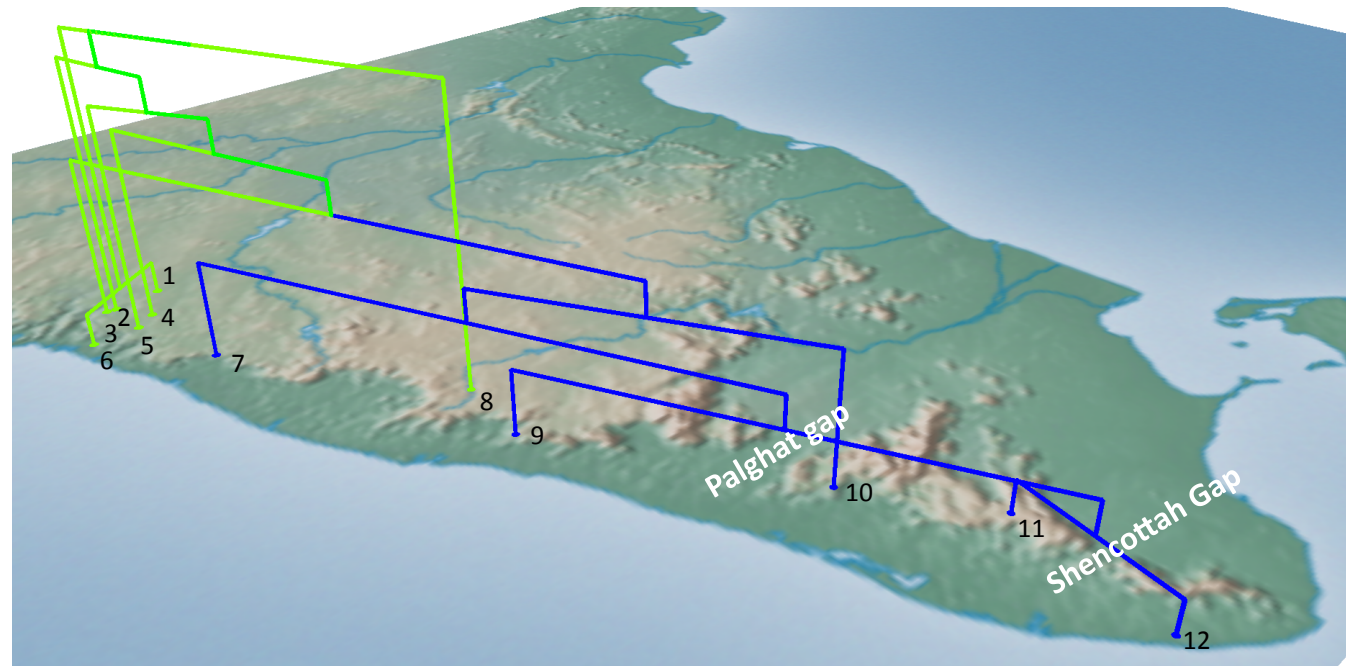

Supplement: Supplementary file 2 [file ece30003-3233-SD2.pdf]

Fig.S3

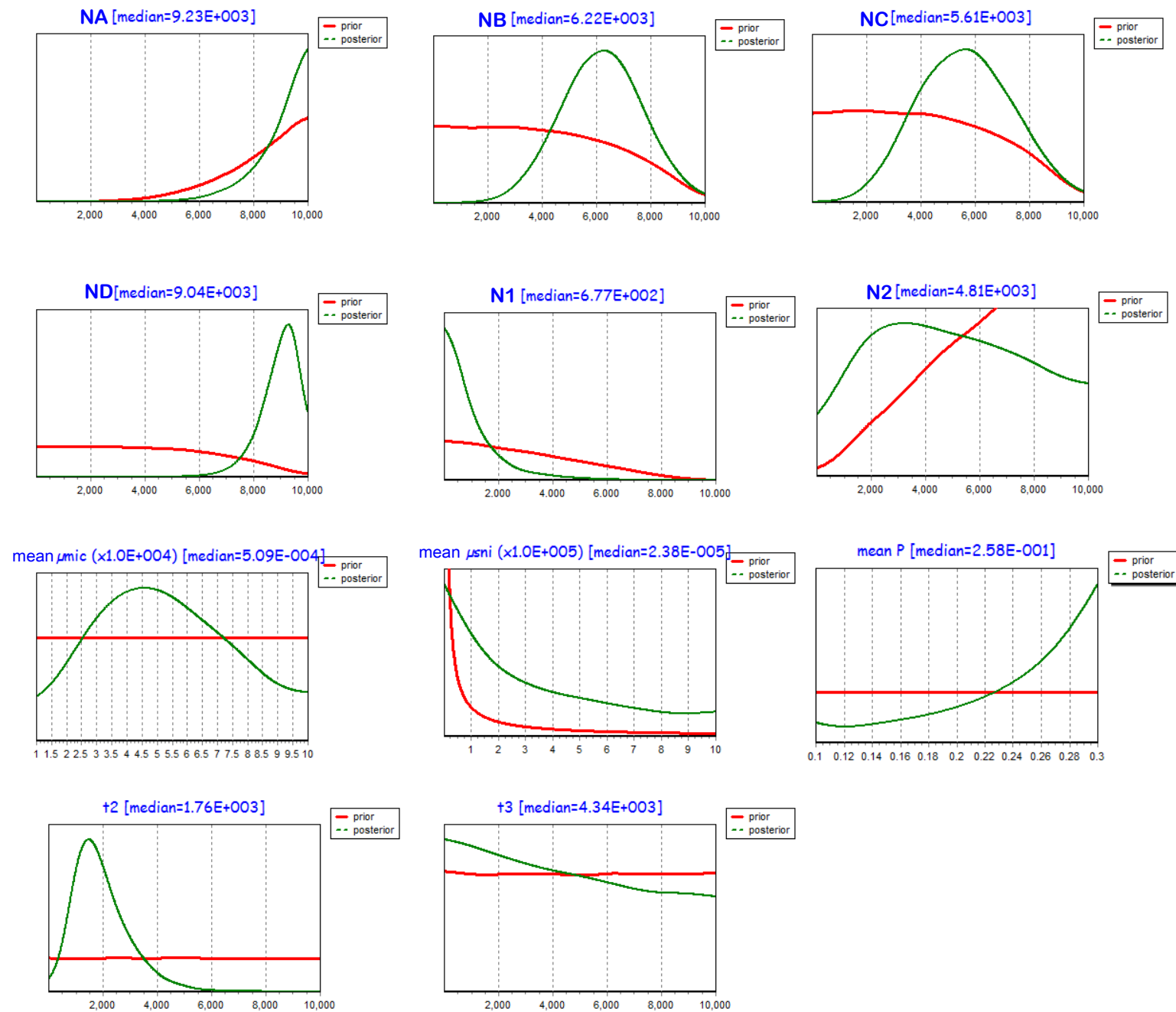

Supplement: Supplementary file 3 [file ece30003-3233-SD3.pdf]

Fig.S4

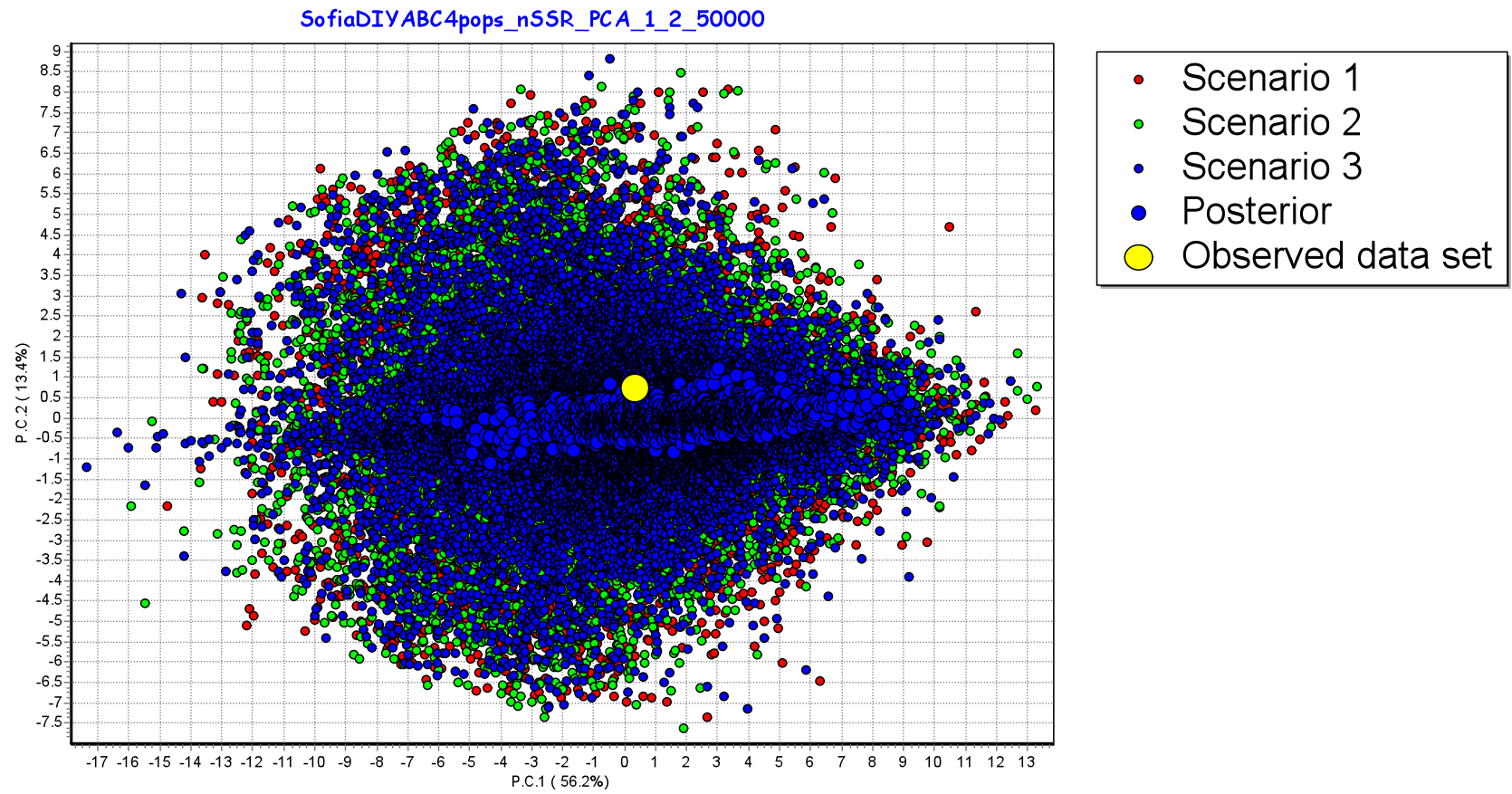

Supplement: Supplementary file 4 [file ece30003-3233-SD4.pdf]
