# Supplementary material for: Genetic structure and demographic history of the endangered tree species Dysoxylum malabaricum (Meliaceae) in Western Ghats, India: implications for conservation in a biodiversity hotspot
Source: Ecol Evol. 2013 Aug 6;3(10):3233–48. doi: 10.1002/ece3.669 (PMC3797473; doi:10.1002/ece3.669)
Supplement: Supplementary file 6 [file ece30003-3233-SD6.docx]

| TableS1. Prior distributions of the parameters used in DIYABC |  |  |
| --- | --- | --- |
| Parameter | Minimum | Maximum |
| *Effective population size* |  |  |
| N_A_ | 10 | 10000 |
| N_B_ | 10 | 10000 |
| N_C_ | 10 | 10000 |
| N_D_ | 10 | 10000 |
| N_1_ | 10 | 10000 |
| N2 | 10 | 10000 |
| *Time scale in generations* |  |  |
| t1 | 1 | 10000 |
| t2 | 1 | 10000 |
| t3 | 1 | 10000 |
| *Admixture* |  |  |
| ra | 0.001 | 0.999 |
| *Mutation model* |  |  |
| Mean mutation rate | 1×10^-4^ | 1×10^-3^ |
| Individual locus mutation rate | 1×10^-5^ | 1×10^-2^ |
| Mean coefficient P | 1×10^-1^ | 3×10^-1^ |
| Individual locus coefficient P | 1×10^-2^ | 9×10^-1^ |
| Mean SNI rate | 1×10^-8^ | 1×10^-4^ |
| Individual locus SNI rate | 1×10^-9^ | 1×10^-3^ |
|  |  |  |

| Table S2. *F_ST_* values per locus with and without correction for null alleles. | | |
| --- | --- | --- |
| Locus | *F_ST_* | *F_ST_* (ENA) |
| Dysmal 1 | 0.13 | 0.12 |
| Dysmal 2 | 0.11 | 0.10 |
| Dysmal 3 | 0.08 | 0.08 |
| Dysmal 7 | 0.06 | 0.06 |
| Dysmal 9 | 0.05 | 0.05 |
| Dysmal 13 | 0.08 | 0.08 |
| Dysmal 14 | 0.13 | 0.13 |
| Dysmal 17 | 0.06 | 0.06 |
| Dysmal 18 | 0.15 | 0.16 |
| Dysmal 22 | 0.04 | 0.04 |
| Dysmal 26 | 0.09 | 0.09 |
|  |  |  |
| Overall | 0.09 | 0.09 |
|  |  |  |
