# Supplementary material for: Genetic structure and demographic history of the endangered tree species Dysoxylum malabaricum (Meliaceae) in Western Ghats, India: implications for conservation in a biodiversity hotspot
Source: Ecol Evol. 2013 Aug 6;3(10):3233–48. doi: 10.1002/ece3.669 (PMC3797473; doi:10.1002/ece3.669)
Supplement: Supplementary file 7 [file ece30003-3233-SD7.docx]

| **Table S3 Distribution of chloroplast haplotypes (alleles in ccmp7) in each of the 12 populations of *Dysoxylum malabaricum*** | | | | | | | | | | | | | |  |  |  |  |  |  |  |  |  |  |  |
| --- | --- | --- | --- | --- | --- | --- | --- | --- | --- | --- | --- | --- | --- | --- | --- | --- | --- | --- | --- | --- | --- | --- | --- | --- |
|  | Haplotype | |  |  |  |  |  |  |  |  |  |  |  |  |  |  |  |  |  |  |  |  |  |  |
|  | 121 | 122 | 123 | 124 | 125 | 126 | 127 | 128 | 129 | 130 | 131 | 132 | 133 | 134 | 135 | 136 | 137 | 138 | 139 | 140 | 141 | 142 | 144 | 145 |
| **1. Yakambi** | 10 | 0 | 0 | 0 | 0 | 2 | 1 | 6 | 2 | 2 | 0 | 2 | 0 | 0 | 0 | 1 | 0 | 0 | 0 | 0 | 0 | 0 | 0 | 0 |
| **2. Jadegadde** | 0 | 0 | 0 | 0 | 3 | 4 | 4 | 2 | 3 | 0 | 2 | 0 | 2 | 1 | 0 | 0 | 0 | 0 | 0 | 1 | 0 | 0 | 0 | 0 |
| **3. Hittalahalli** | 0 | 0 | 8 | 9 | 15 | 1 | 0 | 1 | 3 | 2 | 2 | 0 | 4 | 0 | 1 | 0 | 0 | 1 | 0 | 1 | 0 | 0 | 0 | 0 |
| **4. Navangere** | 3 | 0 | 0 | 0 | 5 | 3 | 0 | 2 | 1 | 18 | 0 | 2 | 2 | 1 | 1 | 0 | 0 | 0 | 0 | 0 | 0 | 0 | 0 | 0 |
| **5. Tavanandi** | 3 | 0 | 0 | 13 | 4 | 0 | 2 | 1 | 7 | 12 | 3 | 1 | 1 | 1 | 0 | 0 | 0 | 0 | 0 | 0 | 0 | 0 | 1 | 1 |
| **6. Sarekoppa** | 0 | 0 | 0 | 0 | 14 | 15 | 5 | 3 | 1 | 1 | 0 | 1 | 1 | 0 | 0 | 1 | 0 | 0 | 0 | 0 | 0 | 0 | 0 | 0 |
| **7. Agumbe** | 0 | 0 | 0 | 0 | 0 | 0 | 0 | 0 | 0 | 0 | 0 | 0 | 0 | 3 | 5 | 1 | 0 | 0 | 0 | 2 | 0 | 1 | 0 | 0 |
| **8. Coorg** | 0 | 1 | 6 | 14 | 1 | 0 | 13 | 1 | 0 | 3 | 2 | 1 | 1 | 0 | 0 | 0 | 0 | 0 | 0 | 0 | 2 | 0 | 0 | 0 |
| **9. Peria** | 0 | 0 | 0 | 0 | 0 | 0 | 0 | 0 | 0 | 0 | 0 | 0 | 0 | 0 | 0 | 6 | 0 | 2 | 0 | 0 | 0 | 0 | 0 | 0 |
| **10. Sholayar** | 0 | 0 | 0 | 0 | 0 | 0 | 0 | 0 | 0 | 0 | 0 | 0 | 0 | 1 | 7 | 8 | 3 | 0 | 0 | 0 | 0 | 0 | 0 | 0 |
| **11. Periyar Tiger Reserve** | 0 | 0 | 0 | 0 | 0 | 0 | 0 | 0 | 0 | 0 | 0 | 0 | 0 | 0 | 1 | 6 | 7 | 2 | 1 | 0 | 0 | 0 | 0 | 0 |
| **12. Arippa** | 0 | 0 | 0 | 0 | 0 | 0 | 0 | 0 | 0 | 0 | 0 | 0 | 0 | 1 | 0 | 6 | 8 | 0 | 0 | 1 | 0 | 0 | 0 | 0 |
|  |  |  |  |  |  |  |  |  |  |  |  |  |  |  |  |  |  |  |  |  |  |  |  |  |

| Table S4. Demographic parameters obtained by DIYABC | | |  |  |  |  |  |
| --- | --- | --- | --- | --- | --- | --- | --- |
| Parameter | mean | median | mode | quantile 2.5% | quantile 5% | quantile 95% | quantile 97.5% |
| N_A_ | 8940 | 9230 | 9960 | 6470 | 6980 | 9950 | 9970 |
| N_B_ | 6190 | 6220 | 6310 | 3310 | 3760 | 8590 | 8990 |
| N_C_ | 5630 | 5610 | 5970 | 2480 | 2910 | 8470 | 8890 |
| N_D_ | 8900 | 9040 | 9290 | 7150 | 7570 | 9770 | 9840 |
| t2 | 1980 | 1760 | 1420 | 567 | 714 | 3930 | 4560 |
| N_1_ | 1020 | 677 | 37 | 38 | 63 | 3210 | 4040 |
| t3 | 4550 | 4340 | 241 | 187 | 372 | 9360 | 9680 |
| N_2_ | 4960 | 4810 | 3360 | 750 | 1070 | 9320 | 9660 |
| Mean mutation rate_SSR | 5.25E-04 | 5.09E-04 | 4.48E-04 | 1.68E-04 | 2.01E-04 | 9.03E-04 | 9.49E-04 |
| Mean P* | 0.252 | 0.27 | 0.3 | 0.113 | 0.141 | 0.3 | 0.3 |
| Mean mutation rate_SNI | 3.20E-05 | 2.38E-05 | 3.00E-08 | 1.20E-07 | 3.10E-07 | 8.95E-05 | 9.53E-05 |
| *the parameter of the geometric distribution to generate multiple stepwise mutations | | | | |  |  |  |
